# ALiEM Blog and Podcast Watch: Procedures in Emergency Medicine

**DOI:** 10.5811/westjem.2017.8.34844

**Published:** 2017-09-26

**Authors:** Nikita Joshi, Eric J. Morley, Taku Taira, Jeremy Branzetti, Andrew Grock

**Affiliations:** * Stanford University, Department of Emergency Medicine, Stanford, California; † Stony Brook School of Medicine, Department of Emergency Medicine, Stony Brook, New York; ‡ LAC + USC Department of Emergency Medicine, Los Angeles, California; § NYU/Bellevue Hospital, Ronald O. Perelman Department of Emergency Medicine, New York, New York; ¶ University of California, Los Angeles, Department of Emergency Medicine, Los Angeles, California

## Abstract

**Introduction:**

The *WestJEM* Blog and Podcast Watch presents high-quality, open-access educational blogs and podcasts in emergency medicine (EM) based on the ongoing Academic Life in EM (ALiEM) Approved Instructional Resources (AIR) and AIR-Professional series. Both series critically appraise resources using an objective scoring rubric. This installment of the Blog and Podcast Watch highlights the topic of procedure emergencies from the AIR Series.

**Methods:**

The AIR Series is a continuously building curriculum that follows the Council of Emergency Medicine Residency Directors’ (CORD) annual testing schedule. For each module, relevant content is collected from the top 50 Social Media Index sites published within the previous 12 months, and scored by eight AIR board members using five equally weighted measurement outcomes: Best Evidence in Emergency Medicine (BEEM) score, accuracy, educational utility, evidence based, and references. Resources scoring ≥30 out of 35 available points receive an AIR label. Resources scoring 27–29 receive an “honorable mention” label if the executive board agrees that the post is accurate and educationally valuable.

**Results:**

A total of 85 blog posts and podcasts were evaluated in June 2016. This report summarizes key educational pearls from the three AIR posts and the 10 Honorable Mentions.

**Conclusion:**

The WestJEM Blog and Podcast Watch series is based on the AIR and AIR-Pro series, which attempts to identify high-quality educational content on open-access blogs and podcasts. This series provides an expert-based, post-publication curation of educational social media content for EM clinicians, with this installment focusing on procedure emergencies within the AIR series.

## BACKGROUND

Despite the rapid rise of social media educational content available through blogs and podcasts in emergency medicine (EM),^1^ identification of quality resources for educators and learners has only received preliminary progress.^2^–^6^ In 2008, the Accreditation Council for Graduate Medical Education endorsed a decrease in synchronous conference experiences for EM residency programs by up to 20% in exchange for asynchronous learning, termed Individualized Interactive Instruction (III).^7^

To address this need, the Academic Life in Emergency Medicine (ALiEM) Approved Instructional Resources (AIR) Series and AIR-Pro Series were created in 2014 and 2015, respectively, to help EM residency programs identify quality online content specifically on social media.^8^,^9^ Using an expert-based, crowd-sourced approach, these two programs identify trustworthy, high-quality, educational blog and podcast content. For the *WestJEM* Blog and Podcast Watch, summaries of these posts are written by the AIR and AIR-Pro Series’ editorial boards.^10^,^11^

This installment from the AIR Series summarizes the highest scoring social media educational resources on EM procedures.

## METHODS

### Topic Identification

The AIR Series is a continuously building curriculum with topics based on the CORD testing schedule (http://www.cordtests.org/) and its monthly topics.

### Inclusion and Exclusion Criteria

A search of the 50 most frequently visited sites per the Social Media Index (SMI)^12^ was conducted for resources relevant to procedure emergencies, published within the previous 12 months. The search, conducted in June 2016, included blog posts and podcasts written in English for scoring by our expert panel.

### Scoring

Extracted posts were scored without blinding by eight reviewers from the AIR Editorial Board, which is comprised of EM core faculty from various U.S. medical institutions. Two of the AIR Editorial Board members, AG and TT, are reviewers for the *Western Journal of Emergency Medicine*. None of the AIR Editorial Board members have conflicts of interest with this publication series. The scoring process allows quality and educational-utility assessment for each blog post and podcast identified. The scoring instrument contains five measurement outcomes using seven-point Likert scales: Best Evidence in Emergency Medicine (BEEM) score, accuracy, educational utility, evidence based, and references (Table 1).^13^ More detailed methods are described in the original description of the AIR Series.^8^,^9^ Board members with any role in the production of a reviewed resource recused him/herself from grading that resource.

### Data Analysis

Resources with a mean evaluator score of ≥ 30 points (out of a maximum of 35) are awarded the AIR label. Resources with a mean score of 27–29 and deemed accurate and educationally valuable by the reviewers are given the “Honorable Mention” label. More in-depth analysis of the methodology of the AIR series can be viewed in the initial article by Lin et al.^9^

## RESULTS

The SMI-50 search yielded 85 blog posts and podcasts relevant to procedures, all of which were filtered and scored. Three AIR and 10 “Honorable mention” posts met our predetermined cut-offs. These 13 posts and podcasts are described below.

### AIR Content

#### 1. Nickson C. Apnoeic Oxygenation. Life in the Fast Lane. (January 10, 2016) AIR http://lifeinthefastlane.com/ccc/apnoeic-oxygenation/

This blog post provides an overview of apneic oxygenation: defining the concept of safe apnea time, describing the relevant physiology, instructing on patient application, and analyzing the published literature.

##### Take-home points

Apneic oxygenation is an adjunct to pre-oxygenation prior to endotracheal intubation that can significantly increase the time before critical arterial desaturation, defined as a SaO2 below 88–90%. Apneic oxygenation can be particularly useful in critically ill patients who are prone to rapid hypoxia with intubation. The ideal method of apneic oxygenation is to provide oxygen via a nasal cannula set at oxygen flow rate of 15L/min oxygen. While it can be initiated at any time during the intubation process, it is ideally started before the administration of an induction agent. If the pre-induction SaO2 is below 95%, positive- pressure ventilation can be used in conjunction with nasal cannula prior to the intubation attempt. Although the literature on apneic oxygenation is both flawed and inconclusive, no studies show harm or desaturation as compared with standard treatment.

#### 2. Weingart S. The Central Line Show Part 1: Avoiding Complications. (August 29, 2015) AIR http://emcrit.org/podcasts/central-line-show/

This blog post focuses on preventing complications from central line placement.

##### Take-home points

The podcast first discusses unrecognized arterial line placement. To avoid this complication, Dr. Weingart advocates for confirmation of venous puncture prior to dilation. This is especially important in non-crash cases as well as with large-bore hemodialysis catheter placement. He outlines in detail a few confirmation methods including pressure transduction with the wire sheath, and the bubble test (also known as the flush test or rapid atrial swirl sign). He includes videos that demonstrate these methods in full detail. If a central line is inadvertently placed in an artery, he recommends to consult vascular surgery and not to remove the catheter in the subclavian position. He lastly discusses methods to prevent a lost guidewire: deliberate practice, improved training and supervision, and the avoidance of interruptions.

#### 3. Rezaie S. All Vascular Access Episode. REBEL EM. (November 12, 2015) AIR http://rebelem.com/november-2015-rebelcast-all-vascular-access-episode/

This podcast covers two recent publications in vascular access. The first covers intravascular complications of central venous catheter (CVC) access and the second covers ultrasound- (US) vs. landmark-guided peripheral intravenous (IV) access.^14^,^15^

##### Take-home points

Among the three standard sites for CVC placement , the subclavian vein has the lowest risk of infectious complications when compared to internal jugular and femoral vein. In contrast, the femoral vein has the lowest rate of mechanical complications. Patient factors and the clinical scenario should determine which site is most appropriate. Regarding peripheral IV placement in patients with no palpable visible veins, the reviewed paper supports the use of US guidance over traditional landmark techniques. In patients with visible or palpable peripheral veins, the traditional landmark technique is quicker with better success rates.

#### 4. Nickson C. Preoxygenation. Life in the Fast Lane. (March 15, 2016) Honorable Mention http://lifeinthefastlane.com/ccc/preoxygenation/

This blog post reviews the methods, techniques, troubleshooting, and complications of pre-oxygenation prior to attempting endotracheal intubation.

##### Take-home points

The primary mechanism of pre-oxygenation is a process called denitrogenation, in which the nitrogen in the lungs is replaced with oxygen. In a healthy, fully pre-oxygenated patient, the safe apneic time is approximately eight minutes. In comparison the safe apneic time is as short as one minute in the patient who is not pre-oxygenated. For patients with SpO2 > 95% and adequate respiratory drive, a non-rebreather at 15L/min is usually effective for pre-oxygenation. High-flow nasal cannula for a minimum of three minutes may be an acceptable, and perhaps superior, alternative. For hypoxic patients, a bag-valve mask (BVM) with PEEP valve at 15L/min should be used. Positive pressure ventilation can improve pre-oxygenation in patients with inadequate respiratory drive. Reasons for inadequate pre-oxygenation include decreased preparatory time, poor mask seal, uncooperative patient, airway obstruction, poor respiratory reserve, and shunt physiology. Pre-oxygenation can be combined with apneic oxygenation.

#### 5. Rezaie S. REBEL Cast: The All Thoracotomy Episode (October 8, 2015) Honorable Mention http://rebelem.com/october-2015-rebelcast-the-all-thoracotomy-episode/

This blog and podcast reviews two articles discussing the factors that influence successful outcomes after resuscitative thoracotomy (RT).^16^,^17^

##### Take-home points

The first paper is a single center, observational study of survival after RT. In this study, every patient who survived or became an organ donor after RT had cardiac motion on Focused Assessment with Sonography in Trauma (FAST) exam. There were no survivors after RT in patients with no cardiac motion or pericardial effusion on FAST. Importantly, this data came from an institution that regularly performs RT. Thus, it likely represents a best-case scenario. The second article is a systematic review and meta-analysis that investigated factors that influence successful RT after blunt traumatic arrest. The article concludes that RT is not recommended for blunt trauma patients who have neither vital signs at any time after injury nor non-survivable head injuries. However, RT should be considered in patients who arrest upon arrival to the ED or have less than 15 minutes of CPR.

#### 6. Morgenstern J. Neonatal (Newborn) Resuscitation 2015 Update. First10EM. (November 2, 2015) Honorable Mention https://first10em.com/2015/11/02/neonatal-resuscitation-2015/

This is a blog post that reviews neonatal resuscitation and new recommendations from the 2015 International Liaison Committee on Resuscitation, American Heart Association, and European Resuscitation Council (ILCOR, AHA, and ERC) guidelines.^18^,^19^

##### Take-home points

The most important guideline updates are as follows: routine intubation and suctioning is no longer required for meconium; heart rate is measured using electrocardiogram leads and not umbilical cord palpation; and positive pressure ventilation can be used for respiratory distress or persistent cyanosis. The author also describes his protocol for neonatal resuscitation. Prior to neonate arrival, call for more physician help, assemble sufficient staff, and ready the necessary equipment including the warmer, medications, and appropriately-sized lines and endotracheal intubation supplies. Once present, the neonate requires appropriate positioning and warming. In premature infants < 28 weeks, use plastic materials to wrap the child to maintain warmth as towel drying can damage the fragile skin. If the neonate is > 28 weeks, proceed to rigorous stimulation. The initial assessment includes term, tone, and breathing and crying. After the initial interventions, reevaluate the heart rate every 30 seconds and intervene if there is no improvement by escalating to BVM and then to chest compressions. Consider ventilation problems from obstruction or underlying lung disease, cardiac pathology, shock, or sepsis.

#### 7. Horeczko T. PEM Playbook – Adventures in RSI. EM Docs. (November 1, 2016) Honorable Mention http://pemplaybook.org/podcast/adventures-in-rsi/

This blog and podcast uses various clinical scenarios to discuss pediatric airway management with a focus on advanced decision-making.

##### Take-home points

The blog presents an in-depth discussion about choice and dosing of induction and paralytic agents for four cases: sepsis, trauma, congenital cardiac disease, and status epilepticus. The author emphasizes that in managing critically ill patients the provider should resuscitate prior to intubation, increase the paralytic dose, and decrease the sedative dose. Providers should also prepare for post-intubation care prior to intubation. Lastly, the relative advantages and disadvantages of ketamine, etomidate, rocuronium, succinylcholine, and propofol are reviewed in each of these clinical scenarios. For sepsis, this post recommends ketamine with etomidate as second line. For trauma, the induction agent depends on the clinical scenario. For cardiogenic shock in a “blue baby,” ketamine is recommended, but for a “pink baby” etomidate is preferred. Lastly, for status epilepticus, propofol or ketamine can be used.

#### 8. Rezaie S. Complications of Procedural Sedation. REBEL EM. (February 22, 2016) Honorable Mention http://rebelem.com/complications-of-procedural-sedation/

This blog post reviews a systematic review and meta-analysis of the incidence of adverse events during procedural sedation.^20^

##### Take-home points

In the 55 articles (25 randomized control trials and 30 observational studies) included in the review, the most common adverse events from procedural sedation were hypoxia, vomiting, hypotension, and apnea. Severe adverse events including aspiration, laryngospasm, and intubation were extremely rare. The post praised the methodology of the reviewed study, citing the vigorous search strategy, which included eight electronic databases, adherence to PRISMA (Preferred Reporting Items for Systematic Reviews) guidelines, and a high level of inter-observer agreement among the reviewers. These results can be used for enhanced shared decision-making with patients, which is further facilitated by a pocket card published with the meta-analysis.

#### 9. Downham J. 6 Ways to Be Better with the Bag-Valve Mask. Critical Care Practitioner. (June 6, 2016) Honorable Mention http://www.jonathandownham.com/6-ways-to-be-better-with-the-bag-valve-mask/

This blog post reviews BVM ventilation, common pitfalls, and strategies to maximize its success.

##### Take-home points

BVM ventilation is both a life-saving technique and a learned skill. As a skill, like intubation it requires practice and refinement to optimize. The three major errors in BVM ventilation are poor positioning, poor mask seal, and poor ventilation. Proper ventilation can be maximized by placing the head in the sniffing position, raising the head to align the ear to sternal notch, and placing both an oropharyngeal airway and a nasopharyngeal airway. Poor mask seal is best resolved with the two-handed thenar technique instead of the EC-clamp method. Apneic oxygenation, with a nasal cannula at 15 L/min, can be used in addition to BVM in patients who are difficult to bag.

#### 10. Kilian M and Helman A. Episode 76 – Pediatric Procedural Sedation. EM Cases. (January 2016) Honorable Mention https://emergencymedicinecases.com/pediatric-procedural-sedation/

This blog post reviews the management of pediatric procedural sedation including pre-procedural planning, medication review, and post-procedural assessments.

##### Take-home points

The clinician should consider all procedural sedation options prior to starting the sedation. Possible options are upfront pain management with intranasal (IN) fentanyl and/or an oral analgesic early in the patient’s ED course; ample use of distracting techniques; keeping familiar faces around the child; and encouraging family presence during the sedation. Based on current evidence, there is no clear indication to delay an urgent procedure because of time since the last meal.

The blog additionally reviews the relative risks and benefits of ketamine, etomidate, propofol, and nitrous oxide. The authors recommend the use of IV and intramuscular ketamine whenever possible and strongly urge against fentanyl with midazolam. In addition to these drugs, which are commonly used for painful procedures, the authors recommend IN or oral midazolam for non-painful procedures such as diagnostic imaging. The authors additionally recommend the use of sucrose for infants undergoing lumbar puncture. With regard to post-sedation management, the authors emphasize the importance of clinical parameters over strict time guidelines. The patient should be monitored until he is able to tolerate oral intake and at baseline functional status.

#### 11. Turchiano M. Procedural Sedation and Analgesia Resources. (July 15, 2016) Honorable Mention http://coreem.net/core/procedural-sedation-and-analgesia-resources/

This blog posts includes an extensive three-part video series that covers procedural sedation preparation, mitigation of harm, and sedative agents.

##### Take-home points

The author emphasizes the need for an organized and systematic approach to the preparation and management of procedural sedation and analgesia (PSA). In preparing for PSA, the authors recommend clinicians use the included checklist to systematically review the appropriateness of the sedation method, medication, monitoring, and material preparation. Although hypoxia, hypotension, and vomiting can occur during PSA, the authors emphasize the importance of awareness and evaluation of obstruction and hypoventilation as these are by far the most common complications. To monitor for obstruction and hypoventilation, the author recommends the routine use of real-time waveform capnography, with an emphasis on monitoring the capnograph and not the absolute ETCO2 level. Additionally, the author stresses the need for a systematic approach to the management of hypoventilation, which emphasizes airway maneuvers over immediate BVM ventilation.

Additionally, the videos contain an extensive discussion of the pros and cons of common sedative agents. Overall, the author recommends the use of propofol for procedures that are brief and/or require profound muscle relaxation, and ketamine for procedures that are longer and/or in children.

#### 12. Vetter N and Sturm J. Procedural Sedation Errors in the Emergency Department. (May 11, 2016) Honorable Mention http://www.emdocs.net/top-10-errors-of-procedural-sedation-in-the-emergency-department/

This post highlights 10 common errors during emergency department PSA and provides strategies for mitigating them.

##### Take-home points

Based on both the best available evidence and the American College of Emergency Physicians’ 2013 clinical policy, clinicians should not delay sedation for an urgent procedure because of a recent meal. While severe complications from PSA are rare, providers should nonetheless prepare the necessary airway equipment in advance, including suction, airway adjuncts, and intubation equipment. For the management of hypoventilation, clinicians should use a stepwise approach starting with the cessation of further sedatives and incorporation of airway positioning maneuvers before the use of a BVM. The authors emphasize the importance of maintaining proper ventilation via capnography, as this will identify hypoventilation earlier than hypoxemia via pulse oximeter. The authors recommend ketamine for PSA, but caution clinicians to be prepared to prevent and treat emergent reactions. In addition, as propofol requires a more frequent dosing due to lack of tissue accumulation, clinicians can provide more generous upfront dosing (1 mg/kg), and then revert to maintenance doses (0.5mg/kg) every 5–10 minutes. Lastly, PSA medications dosing should start low and re-dosing should occur less frequently in elderly patients because of their increased sensitivity to PSA medications.

#### 13. Kurkowski E. Ultrasound-Guided Pericardiocentesis. Core EM. (July 22, 2015) Honorable Mention http://coreem.net/core/ultrasound-guided-pericardiocentesis/

This post reviews the clinical presentation, diagnosis, and management of pericardial tamponade as well as reviewing both ultrasound-guided and landmark-based pericardiocentesis.

##### Take Home Points

Pericardial tamponade commonly presents with dyspnea or decreased exercise tolerance, tachycardia, and hypotension. The treatment for atraumatic pericardial tamponade is pericardiocentesis, which can be performed either with ultrasound guidance or through a landmark-based approach. This post recommends an ultrasound-guided approach because it allows for real-time visualization of both the effusion and the needle insertion into the pericardium. The parasternal approach may be preferred due to the shorter distance from skin and decreased chance of damaging interposed organs. The authors cite a case series of nine patients to provide a recommendation that use of ultrasound is preferred because of the decreased risk of injury and reduction for the need for more invasive surgical drainage.

## CONCLUSION

The ALiEM Blog and Podcast Watch series serves to identify educational quality blogs and podcasts for EM clinicians through its expert panel using an objective scoring instrument. These social media resources are currently curated in the ALiEM AIR and AIR-Pro Series, originally created to address EM residency needs. These resources are herein shared and summarized to help clinicians filter the rapidly published multitude of blog posts and podcasts. Limitations include that the search only includes content produced within the previous 12 months from the top 50 SMI sites. While these lists are by no means a comprehensive analysis of the entire Internet for these topics, this series provides a post-publication accreditation and curation of recent, online content to identify and recommend high-quality educational social media content for the EM clinician. The other limitation is that the SMI score, which is the initial search criteria, is based upon an impact score and is not a quality indicator itself. Based upon this, it is possible that blog posts and podcasts that would meet the quality and educational marker could be missed. In addition, our scoring cut-offs of 30 and 27 were based on a consensus from the AIR series executive board and includes the highest scoring 20% of blog posts reviewed.

## Figures and Tables

**Table:** Approved Instructional Resources scoring instrument for blog and podcast content with the maximum score being 35 points.

Tier 1: BEEM rater scale	Score	Tier 2: content accuracy	Score	Tier 3: educational utility	Score	Tier 4: evidence-based medicine	Score	Tier 5: referenced	Score
Assuming that the results of this article are valid, how much does this article impact on EM clinical practice?		Do you have any concerns about the accuracy of the data presented or conclusions of this article?		Are there useful educational pearls in this article for senior residents?		Does this article reflect evidence -based medicine (EBM)?		Are the authors and literature clearly cited?	
Useless information	1	Yes, many concerns from many inaccuracies	1	Not required knowledge for a competent EP	1	Not EBM based; only expert opinion	1	No	1
Not really interesting, not really new, changes nothing	2		2		2		2		2
Interesting and new, but doesn’t change practice	3	Yes, a major concern about few inaccuracies	3	Yes, but there are only a few (1–2) educational pearls that will make the EP a better practitioner to know or multiple (>=3) educational pearls that are interesting or potentially useful, but rarely required or helpful for the daily practice of an EP	3	Minimally EBM based	3		3
Interesting and new, has the potential to change practice	4		4		4		4	Yes, authors and general references are listed (but no in-line references)	4
New and important: this would probably change practice for some EPs	5	Minimal concerns over minor inaccuracies	5	Yes, there are several (>=3) educational pearls that will make the EP a better practitioner to know, or a few (1–2) every competent EP must know in their practice	5	Mostly EBM based	5		5
New and important: this would change practice for most EPs	6		6		6		6		6
This is a “must know” for EPs	7	No concerns over inaccuracies	7	Yes, there are multiple educational pearls that every competent EP must know in their practice	7	Yes, exclusively EBM based	7	Yes, authors and in-line references are provided	7

*BEEM*, Best Evidence in Emergency Medicine; *EP*, emergency physician; *EBM*, evidence-based medicine.
